# Comparison of the Efficacy of Adjunctive Aids After Scaling and Root Planing in Patients With Chronic Periodontitis

**DOI:** 10.7759/cureus.95358

**Published:** 2025-10-24

**Authors:** Shivani G, Vidya Pranati, Rekha R Koduganti, Himabindu Gireddy, Suhail Mir, Suman S

**Affiliations:** 1 Department of Periodontics, Panineeya Institute of Dental Sciences, Hyderabad, IND

**Keywords:** chronic periodontitis, clinical parameters, injectable platelet rich fibrin, scaling and rootplaning, silver nano particles

## Abstract

Background: Periodontitis is a chronic inflammatory disease marked by periodontal tissue destruction and alveolar bone loss due to microbial biofilms and an exaggerated host immune response. Local drug delivery (LDD) systems are introduced to enhance site-specific therapeutic efficacy while minimizing systemic effects. Nanoparticle-based gels offer sustained drug release and improved tissue penetration, whereas injectable platelet-rich fibrin (i-PRF), an autologous biologic, is rich in growth factors and promotes periodontal regeneration.

Aim: To assess the efficacy of silver nanoparticle (AgNP) gel and i-PRF as adjuncts to nonsurgical periodontal therapy.

Methods: This was an interventional single-arm study. Thirty systemically healthy patients with chronic periodontitis were recruited, each group comprising 10 individuals. Investigator (KRR) examined the patients at baseline for the clinical parameters and again after 24 h to check the reproducibility of the measurements. The calibrations were considered reproducible when the baseline and 24‑h measurements tallied within 1 mm, 95% of the time. The samples were screened and randomly assigned in sealed envelopes by investigator KRR into Groups A, B, and C. Treatment was performed by investigator SG. Investigator (SG) performed scaling and root planing (SRP) at the second visit (after 24 h), following which, patients were administered the local delivery systems in the test groups A (SRP+AgNP gel) and B (SRP+i-PRF), whereas no LDD was administered in Group C (control group). The clinical parameters, gingival index (GI), pocket probing depth (PPD), and clinical attachment level (CAL) were assessed at baseline and after six weeks.

Results: Intragroup comparison was performed using the paired t-test. One-way analysis of variance (ANOVA) was used to compare the mean values of the clinical parameters between the groups. Post hoc Tukey test was applied to evaluate the differences between groups. A p-value of <0.05 was considered statistically significant. At six weeks, all three groups showed statistically significant improvements in the clinical variables, compared to baseline (p < 0.05). The AgNP gel group (Group A) demonstrated the greatest reduction in GI (1.44 ± 0.33) and PPD (3.9 ± 0.74 mm), while the i-PRF group (Group B) showed superior CAL gain (4.2 ± 0.86 mm). Inter-group comparison revealed a statistically significant difference in GI (p = 0.046), favoring the adjunctive therapies over SRP alone. No adverse effects were reported throughout the study.

Conclusion: Both AgNP gel and i-PRF effectively enhanced periodontal healing when used after SRP. AgNP showed stronger antimicrobial action, while i-PRF promoted better tissue regeneration, highlighting their potential as valuable adjuncts to nonsurgical periodontal therapy.

## Introduction

Periodontitis is an inflammatory disease left untreated, can progressively cause loss of teeth. It is primarily initiated by microbial plaque biofilm that triggers a host-mediated inflammatory response, leading to clinical signs such as gingival inflammation, bleeding on probing, periodontal pocket formation, and eventual tooth mobility or loss [1]. Standard periodontal therapy begins with full mouth cleaning, which involves mechanical debridement to eliminate subgingival biofilms and calculus. Although SRP remains the cornerstone of non-surgical periodontal treatment, its efficacy can be limited, especially in deep pockets and areas with complex root anatomy where residual pathogens may persist [2].

To enhance the effectiveness of scaling and root planing (SRP) and improve healing outcomes, several additional therapies have been explored. Among these, silver nanoparticles (AgNPs) have emerged as promising agents due to their potent antimicrobial, anti-inflammatory, and wound-healing properties. AgNPs exert their bactericidal effects by disrupting microbial cell membranes, generating reactive oxygen species, and interfering with DNA replication. These mechanisms make them highly effective against a broad range of putative periodontal pathogens [2]. Additionally, AgNPs are biocompatible, available commercially as pastes or gels for local application in periodontal pockets, potentially reducing bacterial load and promoting faster tissue regeneration [1]. Injectable platelet-rich fibrin (i-PRF) represents a novel biologic approach to enhance periodontal regeneration. It is a second-generation autologous platelet concentrate obtained through low-speed centrifugation without anticoagulants. It is rich in platelets, leukocytes, fibrin, and a host of growth factors such as platelet-derived growth factor (PDGF), transforming growth factor-beta (TGF-β), and vascular endothelial growth factor (VEGF), all of which play pivotal roles in angiogenesis, extracellular matrix remodeling, and regeneration [3]. Unlike traditional PRF, i-PRF is in a liquid form and can be easily injected into periodontal pockets or combined with biomaterials, offering better adaptability and a sustained supply of growth factors over time [4]. Given the unique properties of both silver nanoparticles and i-PRF, their clinical effectiveness as adjuncts to SRP in treating chronic periodontitis has been assessed in this study. Improvements in the gingival index (GI), pocket probing depth (PPD), and clinical attachment level (CAL) after treatment with these agents when used as adjunctive aids after scaling have been comparatively studied.

## Materials and methods

This single-centered, randomized, controlled parallel-design study was carried out in the Department of Periodontics at a dental hospital setting following CONSORT guidelines, after being permitted by the Institutional Ethical Committee and Review Board, Panineeya Institute of Dental Sciences & Research Centre, Hyderabad (Approval No.: PMVIDS&RC/IEC/PERIO/PR/627-24). The study population was selected from the subjects visiting the outpatient section of the Department of Periodontics at Panineeya Dental College and Hospitals, Hyderabad, between April 2024 and March 2025.

The inclusion and exclusion criteria were as follows: (i) Patients aged between 25-55 years, with pocket depths >5 mm, who had not received periodontal treatment within the preceding six months of the study, were included; (ii) Patients who were on antibiotics/antiinflammatory drugs in the last 3 months, pregnant and lactating females, medically compromised patients, smokers, tobacco chewers, alcoholics, and patients who were allergic to the drugs were excluded from the study.

**Figure 1:**
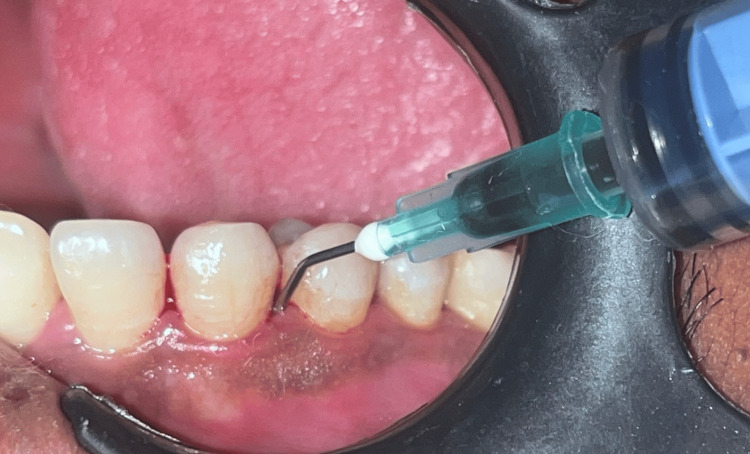
Administration of silvernitrate nanoparticle gel (AgNP) into the periodontal pocket

This was followed by clinical data collection using a Williams periodontal probe. Following phase I therapy, periodontal pockets were irrigated with copious saline to flush the disrupted biofilm and calculus out of the pocket environment. The samples were screened and randomly assigned in sealed envelopes by investigator KRR into Groups A, B, and C. Treatment was performed by investigator SG. The patients in Group A received AgNP gel (commercially available from PerioBiologics LLP, Hyderabad, Telangana, India) as local drug delivery into the pockets after scaling (Figure 1).

Patients in Group B received i-PRF after scaling, and patients in Group C underwent full mouth debridement only.

Collection of i-PRF

**Figure 2:**
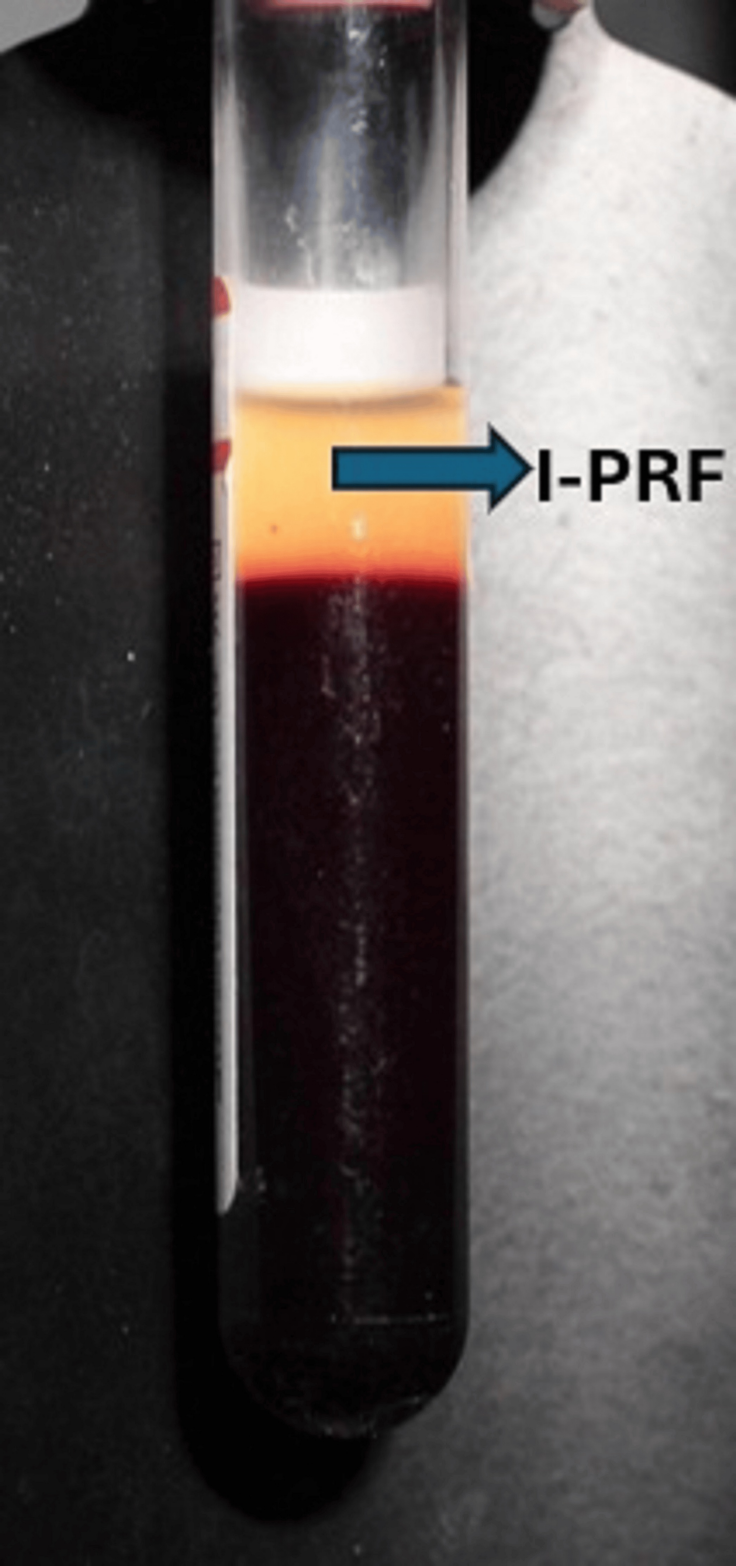
Injectable PRF (i-PRF) was procured after centrifugation

The i-PRF was prepared by the same operator according to the protocol developed by Miron and Choukron in 2017 [3] using a centrifuge (Figure 2).

**Figure 3:**
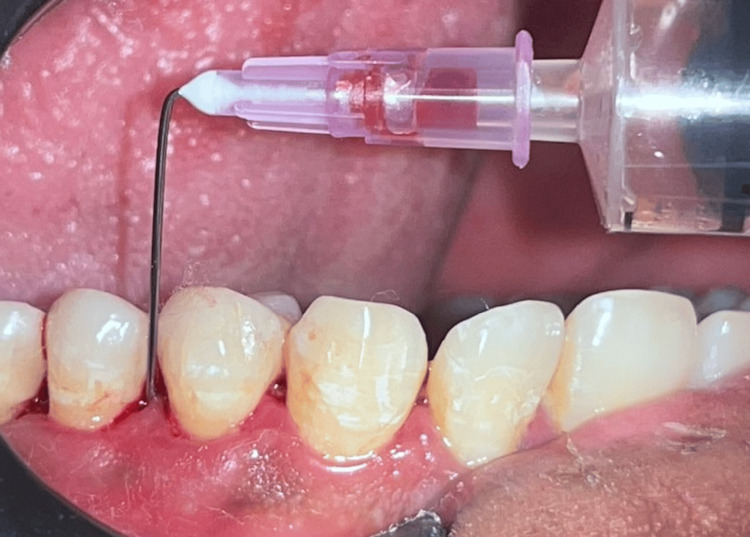
Injectable PRF (i-PRF) being administered into the periodontal pocket

After centrifugation, the top PRF layer was aspirated into a 2-mL syringe and maintained in liquid consistency for about three to five minutes, and used as a local drug delivery agent (LDD) in Group B (Figure 3).

After insertion of the drug locally in the test groups, the region was secured with the periodontal dressing.

Postoperative protocol

All the patients refrained from chewing hard and sticky food, flossing on the treated side, and were instructed not to disturb the area with the tongue, finger, or a toothpick. For the removal of the periodontal dressing, the patients reported after one week. The clinical parameters were reassessed at six weeks and recorded.

## Results

All three groups, Group A (SRP + AgNP Gel), Group B (SRP + i-PRF), and Group C (SRP only), showed statistically significant improvements in clinical parameters from baseline to the six-week follow-up (p < 0.05), validating the benefits of nonsurgical periodontal therapy. At baseline, the mean GI scores were comparable across the groups: 2.21 ± 0.39 in Group A, 2.24 ± 0.59 in Group B, and 2.16 ± 0.28 in Group C. After six weeks, a significant reduction was observed in all groups, with Group A exhibiting the greatest improvement (1.44 ± 0.33), followed by Group B (1.52 ± 0.44), and Group C (1.53 ± 0.42). The PPD also showed a marked decrease across all groups. Group A recorded a reduction from 5.2 ± 0.86 mm at baseline to 3.9 ± 0.74 mm at six weeks, while Group B showed a decrease from 5.9 ± 0.99 mm to 4.05 ± 0.93 mm, and Group C from 4.9 ± 0.97 mm to 3.95 ± 0.91 mm. The reduction in PPD was statistically significant within each group (p < 0.05). Similarly, CAL improved significantly within all groups six weeks postoperatively. Group A demonstrated a gain from 5.3 ± 0.82 mm to 4.0 ± 0.68 mm, Group B from 5.35 ± 0.94 mm to 4.2 ± 0.86 mm, and Group C from 5.0 ± 0.98 mm to 4.1 ± 0.98 mm (p < 0.05 for all) (Table 1).

**Table 1:** Intra-group comparison of clinical parameters at baseline and six weeks *statistically significant SRP: scaling and root planing; AgNP: silver nanoparticle gel; i-PRF: injectable platelet-rich fibrin

Group	Time point	Gingival index (GI)	Probing pocket depth (PPD) (mm)	Clinical attachment level (CAL) (mm)
Group A( SRP + AgNP gel)	Baseline	2.21 ± 0.39	5.2 ± 0.86	5.3 ± 0.82
	6 Weeks	1.44 ± 0.33*	3.9 ± 0.74*	4.0 ± 0.68*
Group B (SRP + i-PRF)	Baseline	2.24 ± 0.59	5.9 ± 0.99	5.35 ± 0.94
	6 Weeks	1.52 ± 0.44*	4.05 ± 0.93*	4.2 ± 0.86*
Group C (SRP only)	Baseline	2.16 ± 0.28	4.9 ± 0.97	5.0 ± 0.98
	6 Weeks	1.53 ± 0.42*	3.95 ± 0.91*	4.1 ± 0.98*

While all groups showed significant intra-group improvements, comparative analysis suggested that Group A (AgNP Gel) was more effective in early reduction of gingival inflammation, as reflected by the greater reduction in GI, while Group B (i-PRF) achieved a higher gain in CAL, indicating its regenerative potential. The control group (Group C) also showed improvements, but these were generally less pronounced compared to Groups A and B (Table 2). 

**Table 2:** Inter-group comparison of clinical parameters at baseline and six weeks *statistically significant NS: not significant; SRP: scaling and root planing; AgNP: silver nanoparticle gel; i-PRF: injectable platelet-rich fibrin

Clinical parameter	Time point	Group A (SRP + AgNP gel)	Group B (SRP + i-PRF)	Group C (SRP only)	p-value (ANOVA)
Gingival index (GI)	Baseline	2.21 ± 0.39	2.24 ± 0.59	2.16 ± 0.28	0.841 (NS)
	6 Weeks	1.44 ± 0.33a	1.52 ± 0.44a	1.53 ± 0.42b	0.046*
Probing pocket depth (PPD) (mm)	Baseline	5.2 ± 0.86	5.9 ± 0.99	4.9 ± 0.97	0.062 (NS)
	6 Weeks	3.9 ± 0.74a	4.05 ± 0.93a	3.95 ± 0.91a	0.372 (NS)
Clinical attachment level (CAL) (mm)	Baseline	5.3 ± 0.82	5.35 ± 0.94	5.0 ± 0.98	0.573 (NS)
	6 Weeks	4.0 ± 0.68a	4.2 ± 0.86a	4.1 ± 0.98a	0.418 (NS)

No adverse effects were reported in any group, and all patients completed the follow-up period without complications.

At baseline, there were no statistically significant differences among the three groups related to the clinical parameters (p > 0.05). This confirms initial homogeneity across study groups. Intragroup analysis of the clinical parameters at six weeks in all three groups showed statistically significant improvement (p < 0.05). However, inter-group analysis using one-way ANOVA demonstrated a statistically significant difference in GI between the groups (p = 0.046). Post hoc Tukey’s test revealed that Group C (SRP alone) showed significantly higher GI scores compared to the test groups, suggesting that the adjunctive use of AgNP gel and i-PRF resulted in superior reduction in gingival inflammation. No statistically significant inter-group differences were observed in PPD and CAL at six weeks (p > 0.05), although numerically greater reductions were noted in the adjunctive therapy groups (AgNP and i-PRF) when compared to SRP alone. While all the therapies were effective, the addition of either AgNP gel or i-PRF to SRP resulted in greater improvements in gingival inflammation at six weeks. The comparable performance of the test groups further supports the effectiveness of both adjuncts in enhancing nonsurgical periodontal therapy outcomes.

## Discussion

The present study evaluated and compared the clinical effectiveness of subgingivally delivered silver nanoparticle (AgNP) gel and injectable platelet-rich fibrin (i-PRF) as adjuncts to scaling and root planing (SRP) in the management of chronic periodontitis. The results indicated that both adjunctive therapies led to statistically significant improvements in clinical parameters compared to baseline, thereby supporting their beneficial role in nonsurgical periodontal therapy. Group A (AgNP gel) demonstrated a significant early reduction in gingival inflammation and bleeding on probing. This observation is attributed to the potent antimicrobial and anti-inflammatory properties of silver nanoparticles. Due to their small particle size and large surface area, AgNPs are capable of penetrating deep into periodontal pockets, where they exert their bactericidal effects by disrupting microbial membranes, generating reactive oxygen species (ROS), and interfering with microbial DNA replication. These mechanisms effectively reduce the microbial load, particularly targeting putative periodontal pathogens that play key roles in the severity of chronic periodontitis. Preclinical work showed that combining AgNPs with ebselen enhanced intracellular bactericidal activity against Porphyromonas gingivalis and reduced host-cell oxidative damage, suggesting routes to improve AgNP antimicrobial potency while mitigating cytotoxicity, relevant to safety optimization of AgNP gels [5]. A recent split‑mouth randomized clinical trial comparing 0.02% AgNP gel with 2% minocycline gel as adjuncts to SRP reported that both adjuncts produced significantly better clinical and microbiological outcomes than SRP alone, with AgNP performing similarly to minocycline. This supports our finding that AgNP, when used subgingivally, provides rapid antimicrobial/anti‑inflammatory benefit [6]. A clinical study of subgingival AgNP local delivery reported significant clinical improvements and microbial reductions compared with SRP alone/tetracycline, corroborating the efficacy and short-term safety of AgNP gels [7]. Previous studies have highlighted the efficacy of AgNPs in reducing microbial burden and enhancing soft tissue healing, aligning with the findings of the current investigation. A randomized clinical trial of subgingivally delivered propolis nanoparticles (natural-product nanoparticle) showed significant GI, PPD, and relative attachment level (RAL) improvements at one to three months versus SRP + saline, which demonstrates that nanoparticle carriers improve local drug delivery and clinical outcomes, supporting the generalizability of nanoparticle adjuncts (mechanistic parallel to AgNP gels) [8]. An up-to-date review summarized antibacterial, anti-biofilm, and anti-inflammatory properties of metal nanoparticles (including AgNPs), their design considerations, and translational challenges (toxicity, dosing, delivery) that provide mechanistic grounding for why AgNP gels work and cautions for clinical use [9].

In contrast, the i-PRF group exhibited more favorable outcomes in terms of clinical attachment gain and long-term tissue healing. i-PRF, a second-generation autologous platelet concentrate, contains high levels of platelets, leukocytes, fibrin, and a range of growth factors that include platelet-derived growth factor (PDGF), transforming growth factor beta (TGF-β), and vascular endothelial growth factor (VEGF). These bioactive molecules are known to stimulate angiogenesis, promote fibroblast proliferation, enhance extracellular matrix formation, and support tissue regeneration. A meta-analysis concluded that platelet-rich fibrin (including injectable forms) improves PPD and CAL compared with SRP alone and favorably modulates biomarkers (PDGF, VEGF, TGF-β) that align with Group B’s greater CAL gains [10]. i-PRF applied adjunctively reduced MMP-8 levels in gingival crevicular fluid and improved clinical indices, providing biochemical evidence (matrix-remodeling modulation) that explains CAL gain with i-PRF [11]. The injectable nature of i-PRF allows for precise delivery into periodontal pockets and ensures better adaptability to root surfaces, facilitating a prolonged release of growth factors at the treatment site. The findings of the current study are in agreement with existing literature, which emphasizes the regenerative potential of i-PRF in managing periodontal and peri-implant defects. The review of nanoparticle delivery systems (organic and inorganic) emphasized improved drug retention in pockets, sustained release, and higher local concentrations -- mechanisms that help explain why local AgNP gel produced faster GI reductions in the trial [12]. A focused review on silver nanoparticles in dental settings highlights antimicrobial efficacy, wound-healing promotion at low doses, and the need for standardizing formulations and toxicity testing that supports the findings from this study while emphasizing safety research needs [13].

While both adjuncts significantly enhanced the clinical outcomes compared to SRP alone, a comparative analysis suggests that AgNP gel is particularly effective in rapidly controlling infection and inflammation, whereas i-PRF demonstrates superior regenerative capabilities, especially in terms of CAL gain. These differential effects can be attributed to their distinct mechanisms of action, AgNPs being primarily antimicrobial, and i-PRF being biologically regenerative.

The current study's strength lies in its direct clinical comparison of two novel yet mechanistically different adjuncts to SRP in chronic periodontitis. However, it is important to acknowledge certain limitations. The follow-up duration may have been insufficient to fully assess long-term regenerative outcomes, particularly regarding alveolar bone remodeling. Additionally, the absence of microbiological analysis limits the objective quantification of microbial reduction. The use of radiographic or histological assessments in future studies would offer a more comprehensive understanding of the underlying tissue changes and regenerative effects.

Despite these limitations, the study underscores the potential utility of both AgNP gel and i-PRF as effective adjuncts in nonsurgical periodontal therapy. Their use may help overcome the limitations of conventional SRP by enhancing bacterial elimination and promoting soft tissue healing. Further longitudinal studies with larger sample sizes, microbial analysis, and histological evaluations are recommended to validate and expand upon these findings.

## Conclusions

The present study demonstrates that both silver nanoparticle (AgNP) gel and injectable platelet-rich fibrin (i-PRF), when used as adjuncts to scaling and root planing (SRP), result in significant clinical improvements in the management of chronic periodontitis. AgNP gel showed superior early anti-inflammatory and antimicrobial effects, while i-PRF exhibited enhanced regenerative outcomes, particularly in clinical attachment level gain. These findings highlight the distinct yet complementary therapeutic benefits of AgNP and i-PRF, supporting their potential integration into nonsurgical periodontal treatment protocols. However, further long-term studies incorporating microbiological and radiographic assessments are warranted to substantiate and expand upon these results.
